# Quantum computing with neutral atoms

**DOI:** 10.1093/nsr/nwy088

**Published:** 2018-09-10

**Authors:** Mark Saffman

**Affiliations:** Department of Physics, University of Wisconsin-Madison, USA

The power of quantum computation derives from algorithmic methods that exploit the availability of quantum superposition and entanglement to perform computations that are intractable with classical devices. The race is on to develop hardware that will unleash the promise of quantum algorithms. A handful of different types of hardware are currently being developed with the greatest efforts directed at superconducting, quantum-dot, trapped-ion, photonic, and neutral-atom approaches [1]. While all approaches have strengths and weaknesses, and are at different stages of development, the challenge of creating a practical design that can be scaled to a million or more qubits has not yet been met with any of the existing platforms.

One may wonder if we really need a million qubits for a useful quantum computer. The answer depends on our ambition level. On the one hand it is widely believed that a quantum computer with less than 100 reliable qubits will be able to demonstrate a quantum advantage for a well defined computational problem [2]. Demonstration of a quantum computational advantage will generate great excitement in the halls of academia, but will have little broader import. A problem of great practical interest such as *ab initio* design of chemicals for increasing the yield of fertilizers for crop production, to name but one example, may also require just a few hundred qubits, provided computational sequences with circuit depth of 10^10^ or more operations can be reliably performed [3]. However, given the current performance of qubit hardware, which has demonstrated error rates not less than ∼10^−3^, computations with depth 10^10^ will only produce random outcomes of negligible value.

The solution lies in the construction of error-correction circuitry operating on logical qubits, each composed of many physical qubits, which will enable logical error rates many orders of magnitude smaller than physical error rates. Although error correction is remarkably efficient for classical computing devices—Hamming codes detect and correct errors while only requiring roughly 10% overhead in the number of physical bits—quantum error correction is notoriously expensive. New approaches to error correction are the subject of intensive research with one promising direction being the development of codes that are optimized to suppress the dominant error mechanism in a particular physical platform [4,5]. Nevertheless, with physical error rates of 10^−4^, a plausible target for current qubit systems, the overhead required to reach logical error rates of 10^−10^ may be measured in thousands of physical qubits for each logical qubit. Thus a quantum computer able to perform deep calculations on a few hundred logical qubits could require a million physical qubits.

**Figure 1.**
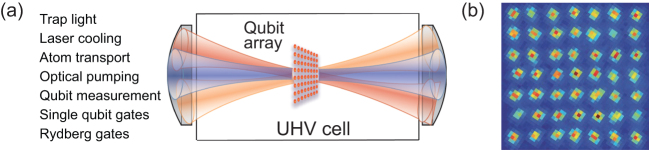
Conceptual picture of a 2D neutral-atom qubit array. a) High-numerical-aperture lenses project several different wavelengths of light for control and measurement of the qubits. Specific choices of optical wavelengths depend on the atomic species. Not shown are optics for initial laser cooling, transport to the array, beam scanning, and imaging for qubit measurement. b) Averaged fluorescence image of 49 atomic qubits on a 2D grid with $3.8 \, {\mu} \rm {m}$ spacing [8].

A consequence of the cost of quantum error correction is that any viable approach to large-scale quantum computing needs to combine high-fidelity quantum logic operations with a capability for integrating large numbers of physical qubits. From this perspective neutral-atom qubits appear particularly promising [6] as they have already demonstrated control of more qubits than any other platform with recent experiments in 1D [7], 2D [8,9], and 3D [10] geometries showing control of up to 50 atomic qubits. Large qubit arrays can potentially be prepared in 1D, 2D, or 3D geometries. The number of proximal qubits increases with the dimensionality, which is advantageous for implementation of error correction code words, and points towards a preference for 2D or 3D arrays. On the other hand, the added overhead of compensating crosstalk incurred when targeting atoms inside a 3D volume makes parallel operations on multiple qubits challenging. A 2D geometry, as shown schematically in Fig. 1, may be an optimal choice since qubit control and measurement can be performed with lasers propagating normal to the plane of the array so that individual qubit sites can be addressed independently. With demonstrated array periods of a few micrometers we require a footprint of less than 1 mm^2^ for 10^4^ qubits.

From a fundamental perspective the prospects for scaling neutral-atom systems are particularly promising due to the exceptionally large ratio between coherent and incoherent coupling rates. Neutral-atom qubits are encoded in hyperfine ground states for which coherence times of the order of 10 s have been demonstrated [10]. This implies a dephasing rate $\gamma \sim 0.1 \, \rm s^{-1}$. The coherent coupling that enables quantum logic operations is switched on by exciting atoms to high-lying Rydberg states with principal quantum number close to 100 [11,12]. Rydberg excited atoms at spacings of a few micrometers in an optical lattice interact coherently with rates $g > 10^9\, \rm s^{-1}$. The ratio between desired coherent coupling and residual incoherent dephasing of *g*/γ ∼ 10^10^ establishes a figure of merit that is enabling for scalability. To our knowledge only trapped ions can claim a similar *g*/γ ratio [13], but without a correspondingly direct path towards controlling thousands of qubits in a single processing unit.

Although the *g*/γ ratio is exceptionally favorable for neutral-atom qubits a complete set of universal gate operations has not yet been demonstrated with high fidelity. Single-qubit gates in large 2D and 3D arrays have reached fidelity ${\mathcal {F}}\sim 0.999$ [8,10]. However, the highest demonstrated fidelity for two-qubit entanglement mediated by Rydberg-state interactions is ${\mathcal {F}}< 0.8$ [6,14,15], a value that is well short of the theoretical limit of ${\mathcal {F}}> 0.9999$ [16,17]. There is healthy optimism within the research community working with neutral-atom qubits that combining improved laser sources with reduced noise, better cooling to reduce motional dephasing, and control of stray electric fields that perturb Rydberg atoms will lead to substantially improved gate fidelity in the near future. Recent results with better filtering of laser phase noise have already demonstrated remarkable improvement in ground–Rydberg entanglement [18].

The outlook for the next few years appears very promising. We anticipate that with advances in lasers, optical lattices for atom trapping, deterministic rearrangement and loading of atoms in multi-dimensional arrays [19,20], and optics for fast beam scanning it will be possible to increase the number of physical qubits in a single neutral-atom module to at least 10^4^. A challenging yet plausible path to a million-qubit processor can then be contemplated by connecting 100 such modules with atom–photon entanglement channels [21]. Beyond the potential for advancing the state of the art in computation, development of techniques for single-atom control inside large multi-atom arrays will enable other applications of quantum science including metrology and time-keeping. Rydberg interactions that are at the heart of a neutral-atom quantum computer may lead to multi-atom entanglement and Heisenberg scaling of atomic-clock precision [22].
